# Diagnosis and management of a urethral prolapse in a 6-year-old girl: a case report

**DOI:** 10.11604/pamj.2021.39.284.30954

**Published:** 2021-08-31

**Authors:** Armand Mayala Ma Mayala, Philippe Lukanu Ngwala, Jean-Pierre Fina Lubaki

**Affiliations:** 1 Department of Family Medicine and Primary Care, Protestant University of Congo, Kinshasa, Congo

**Keywords:** Urethral prolapse, prepubescent girl, case report

## Abstract

Urethral prolapse is a very rare disease, which is most often found in prepubescent girls. It occurs in about one over 5000 girls in the context of a chronic cough or any situation that increases abdominal pressure. It is often associated with diagnostic confusion, which delays management. We report the case of a 6-year-old child brought in urgently by her parents for a minimal genital hemorrhage and presence of a mass protruding from the vulva. In the hypothesis of a urethral prolapse, a medical treatment (conservative) was prescribed. After two weeks, the mass decreased significantly in volume and disappeared completely after 2 months.

## Introduction

Urethral prolapse is the eversion of the urethral mucosa through the urethral meatus [1]. It is a rare condition, first described in 1751 by Morgani [1]. Urethral prolapse remains almost unrecognized due to its low Incidence of about 1/5000 children [2]. It has been reported in several countries in the world and in Africa [2,3]. Unfortunately, to our knowledge, in the Democratic Republic of Congo (DRC), no case has been reported in the literature so far. Urethral prolapse is found in the majority of cases in prepubescent girls, most likely due to the low estrogen concentration in this age group. The pathophysiology of prolapse is not completely understood. It is often related to a weakness of attachment of the internal longitudinal, circular, external oblique muscle fibers and the urethral mucosa. The separation of these planes in situations with increased intra-abdominal pressure could explain the occurrence of mucosal eversion through the urethral meatus [4]. The diagnosis is essentially clinical, the prolapse appears as a mass centered by the urinary meatus, described with a pseudo-tumoral aspect of variable volume that can range from 0.5 to 3cm, pinkish or purplish, edematous, and bleeding readily [5]. Treatment is controversial and involves two modalities. The first is conservative and consists of the application of estrogen-based creams, antiseptic baths, minor analgesics and treatment of urinary tract infection if present [3]. The second modality is surgical either by ligation of the mass around the Foley catheter and the prolapsed part falls out due to lack of irrigation [3], or by excision of the prolapsed mucosa followed by a muco-mucosal suture under general anesthesia [3,5].

## Patient and observation

**Patient information:** FK a 6-year-old, student in 1^st^ year of primary school, living in a peripheral locality of the city of Kinshasa brought by her parents for genital bleeding and the presence of a painful mass on the vulva observed a few hours before the consultation. She is the youngest of four siblings, natural born with a birth weight of 2900 grams; she followed her vaccination schedule and the Bacille Calmette-Guérin (BCG) scar is present. She presents a satisfactory psychomotor development. She was hospitalized three times for malaria and bronchopneumonia. Parents have been reporting chronic constipation for a long time.

**Figure 1:**
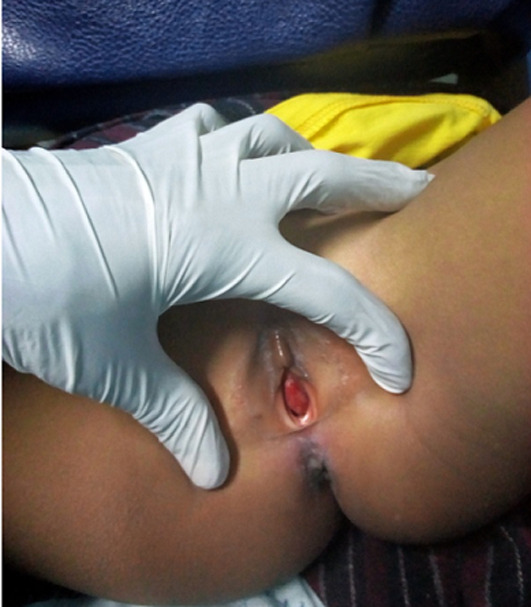
mass protruding from the vulva and centered on the urethral meatus

**Clinical findings:** the general physical examination was normal. At the genital examination, we found a reddish ovale mass of about 1 cm long axis, located just below the clitoris, sensitive to palpation, bleeding on contact. The examination under anaesthesia noted: the labia majora and minora were not oedematous, no abrasions were visualized, the hymen was present, not traumatized and the presence of a rounded mass, more or less firm to palpation, bleeding on contact, revealing the urinary meatus in the center (Figure 1).

**Diagnosis:** urethral prolapse

**Therapeutic interventions:** we opted for a conservative treatment consisting of seat bath with a chloroxylenol-based solution twice a day, application of Baneocin cream twice a day, and estrogen cream twice a day. The girl also received paracetamol as needed for pain. The treatment was followed adequately as reported by the parents. There was no adverse event.

**Figure 2:**
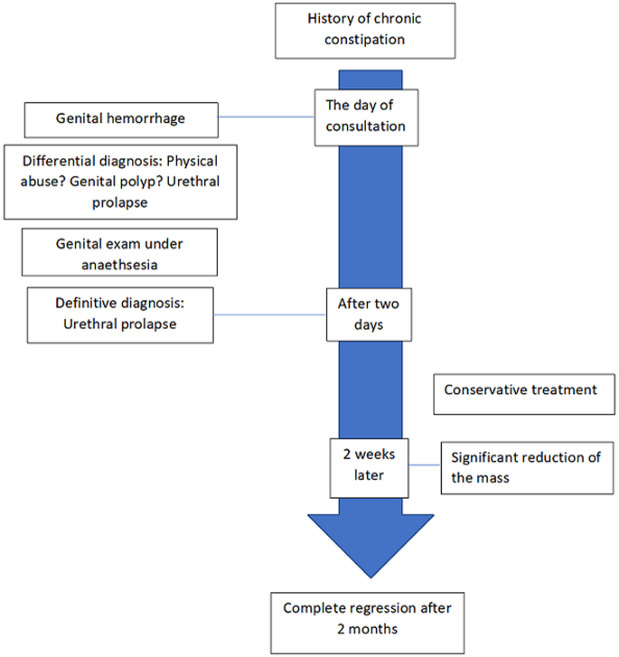
timeline of the disease from the occurrence to complete remission

**Follow-up and outcome of interventions:** conservative treatment was successful for our patient, but the remission was slow (Figure 2). After 2 years of follow-up, we did not observe any recurrence.

**Parents perspectives:** at the beginning, parents were suspecting a sexual abuse of their baby girl. They were reassured by the explanations received from the medical team and the evolution of the disease.

**Informed consent:** the parents give their approval to the publication.

## Discussion

The genital prolapse is a benign disease, the revealing mode can be different according to the cases [6]. In our patient, it was a genital hemorrhage that had soiled the underwear, causing concern to the parents. The parents believed the bleeding was the result of sexual abuse and were relieved by the news of the diagnosis. As factors favoring prolapse, we found, in our patient, a low socio-economic level and chronic constipation; these factors support the evoked mechanism of situations of increased intra-abdominal pressure. Other contributing factors are malnutrition, trauma, urinary and vaginal infections, excess urethral mucosa and physical exertion [5]. Genital hemorrhage in a prepubescent girl is very often the cause of panic among parents and brings a medicolegal dimension requiring the exclusion of sexual abuse. A concern that must be taken into account in the bio-psycho-social care for effective care [7]. Many cases of prolapse have been confused with other pathologies [8,9]. The clinical presentation in our patient also evoked a polyp but the history of constipation, the location of the mass with the urethral meatus in its center, and especially the careful examination under sedation made it possible to retain the hypothesis of urethral prolapse.

## Conclusion

Urethral prolapse is rare and when it concerns the prepubescent girl, raises great concerns about its origin. Its rarity also induces a diagnostic difficulty for the clinician which can delay adequate management.

## References

[1] Paul Lamblin (1903). Le Prolapsus de la muqueuse de l´urètre chez les petites filles. Paris C Naud.

[2] Liu C, Lin Y, Chen X, Li S, Zhu H (2018). Urethral prolapse in prepubertal females: report of seven cases. J Obstet Gynaecol Res.

[3] Okorie CO (2013). Urethral prolapse: contemporary report on a modified ligation over a urethral catheter treatment approach. Nephrourol Mon.

[4] Ballouhey Q, Galinier P, Gryn A, Grimaudo A, Pienkowski C, Fourcade L (2014). Benefits of primary surgical resection for symptomatic urethral prolapse in children. J Pediatr Urol.

[5] Sanda GO, Soumana A, Oumarou H (2012). Le prolapsus muqueux de l´urétre chez la fillette: a propos de 22 cas colligés en dix ans et une revue de la littérature. African J Urol.

[6] Ndour O, Malle K, Fall ALF, Ndoye NA, Nibagora J, Ngom G (2017). Le prolapsus de la muqueuse urétrale chez la fillette: à propos de 12 cas et revue de la littérature. African J Urol.

[7] Smith T, Chauvin-Kimoff L, Baird B, Ornstein A (2020). L´évaluation médicale des enfants prépubères qu´on soupçonne d´être victimes d´une agression sexuelle. Paediatr Child Heal.

[8] Johnson CF (1991). Prolapse of the urethra: confusion of clinical and anatomic characteristics with sexual abuse. Pediatrics.

[9] Shavit I, Solt I (2008). Urethral prolapse misdiagnosed as vaginal bleeding in a premenarchal girl. Eur J Pediatr.

